# Characteristic parameters of photonic nanojets of single dielectric microspheres illuminated by focused broadband radiation

**DOI:** 10.1038/s41598-021-03610-3

**Published:** 2022-01-07

**Authors:** Amartya Mandal, Pragya Tiwari, Paul K. Upputuri, Venkata R. Dantham

**Affiliations:** 1 Department of Physics, Indian Institute of Technology Patna, Daulatpur, Bihar 801103 India; 2 School of Chemical and Biomedical Engineering, Nanyang Technological University, 62 Nanyang Drive, Singapore, 637459 Singapore

**Keywords:** Micro-optics, Sub-wavelength optics

## Abstract

Herein, we report the theoretical investigation on the photonic nanojets (PNJs) of single dielectric microspheres illuminated by focused broadband radiation (polychromatic light) from a Halogen lamp, supercontinuum source, light-emitting diode, and Hg arc lamp. The role of incident beam waist, refractive index of the surrounding medium, and radius of the microsphere on the characteristic parameters such as the electric field intensity enhancement, effective width, and length of the PNJ is studied. Interestingly, the characteristic parameters of the PNJs of solid microspheres obtained for the above-mentioned broadband radiation sources are found close to those observed for the focused monochromatic radiation of wavelengths which are near to the central wavelengths of the sources. Moreover, the characteristic parameters of PNJs of the core–shell microspheres of different thicknesses (*t*) illuminated by polychromatic radiation from most commonly used sources such as Halogen and Hg arc lamps are studied. For each *t* value, a suitable wavelength of monochromatic radiation has been found to generate the PNJ with characteristic parameters which are close to those obtained in the case of polychromatic radiation. We believe that the analytical theory and the theoretical simulations reported here would be useful for researchers who work in the fields such as PNJ assisted photoacoustic spectroscopy, white light nanoscopy, low-coherence phase-shifting interference microscopy, and Mirau interferometry.

## Introduction

Transparent dielectric single microspheres support optical resonances or whispering gallery modes upon the resonant light illumination^1–3^. The quality factor of these resonances is improved significantly when the microspheres are excited with suitable geometries^4–8^. The microspheres are also found to support super-resonances for specific values of size parameters^9,10^. However, narrow-focused and intense electromagnetic beams are generated at the shadow side of these microspheres upon the non-resonant light illumination. These are non-evanescent beams and are named ‘photonic nanojets’ (PNJs)^11–17^. These PNJs are generated from solid/core–shell dielectric microparticles illuminated by a plane wave^18,19^, spherical wave^20^, Gaussian beam^21,22^, Bessel beam^23,24^, etc. The origin of the PNJ is explained based on the forward Mie scattering and focused near-field diffraction^25–27^.

It is to be noted that the PNJs generated by monochromatic light are found useful for enhancing Raman scattering^28–31^, fluorescence signals^32^, nanoparticles sensing^33^, optical data storage^34^, laser surgery^35^, optical transport^36^, two-photon fluorescence^37^, nanolithography^38^, biomedical research^39^ etc. On the other hand, several researchers have generated the PNJs using the focused polychromatic light used for enhancing the backscattering signals of nanoparticles^40^, photoacoustic spectroscopy^41^, white light nanoscopy^42–49^, low-coherence phase-shifting interference microscopy^50^, Mirau interferometry^51^, and super-resolution imaging^52–54^. It is worth mentioning here that several theoretical studies are reported on PNJs of single microspheres illuminated by monochromatic plane and Gaussian waves^18,55–59^. The role of key parameters such as incident beam waist (*ω*_*0*_), refractive index of the surrounding medium (*n*_*m*_), radius (*R*_*s*_), and refractive index (*n*_*s*_) of the microspheres on the characteristic parameters of the PNJs is reported in detail. Few theoretical studies related to the PNJs of single dielectric microspheres under pulsed irradiation (or coherent broadband illumination) are also reported in the literature^60,61^. Under the pulsed irradiation, the PNJs appear to be non-stationary and these are found useful in micromachining beyond the diffraction limit^62^, microscopy^63^, and non-linear optics^64^. However, details of the characteristic parameters of the PNJs generated by focused polychromatic light (or broadband radiation) and the role of *R*_*s*_, *n*_*m,*_ and *ω*_*0*_ on the characteristic parameters of the PNJs generated by broadband radiation are not reported in the literature. Moreover, the differences in the PNJs generated by focused monochromatic light and polychromatic light are not reported. These details would be useful for optimizing the experimental signals obtained using PNJs generated by polychromatic light. Therefore, we have performed a theoretical investigation on the PNJ of single solid and core–shell microspheres illuminated by focused polychromatic light from different sources.

Herein, we report (1) the electric field intensity enhancement (EFIE) distribution inside and outside single dielectric microspheres illuminated by polychromatic light from different light sources such as Hg arc lamp, white LED, supercontinuum (SC) source, and Halogen lamp, (2) the role of *ω*_*0*_, *R*_*s*_, and *n*_*m*_ on the characteristic parameters of PNJs obtained for polychromatic illumination, (3) the characteristic parameters of the PNJs of single microspheres illuminated by monochromatic light, (4) the EFIE distribution inside and outside core–shell microspheres under monochromatic and polychromatic illumination.

## Theoretical aspects

**Figure 1:**
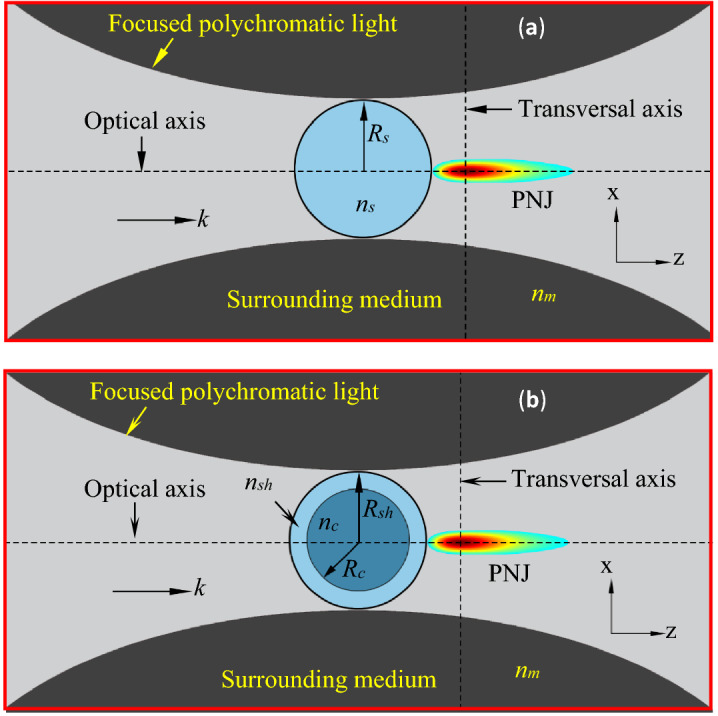
Illustration of PNJs generated by single solid microsphere (panel a) and core–shell microsphere (panel b) under focused polychromatic illumination. Here *R*_*s*_, *R*_*c*_, and *R*_*sh*_ are the radii of the solid microsphere, core, and shell, respectively. *n*_*s*_, *n*_*c*_, *n*_*sh*_, and *n*_*m*_ are the refractive indices of the solid microsphere, core, shell, and surrounding medium, respectively.

Recently, we have developed an analytical theory for estimating the electric field enhancement inside and outside single core–shell dielectric microspheres illuminated by focused monochromatic light^59^. Using this theory, the characteristic parameters of the PNJs generated by core–shell microspheres of different sizes and refractive indices are estimated. In the present study, the analytical theory has been extended to study the characteristic parameters of the PNJs of single solid/core–shell microspheres illuminated by focused polychromatic light (Fig. 1) from different sources.

The final expressions for plotting the EFIE in the surrounding medium (*η*_*m*_), core (*η*_*c*_), and shell (*η*_*sh*_) are given below.

1 $$\eta_{m} = \mathop \Sigma \limits_{p = 1}^{k} I_{m} (r,\theta ,\phi ,\lambda_{p} )$$

2 $$\eta_{c} = \mathop \Sigma \limits_{p = 1}^{k} I_{c} (r,\theta ,\phi ,\lambda_{p} )$$

3 $$\eta_{sh} = \mathop \Sigma \limits_{p = 1}^{k} I_{sh} (r,\theta ,\phi ,\lambda_{p} )$$

4 $$I_{m} (r,\theta ,\phi ,\lambda_{p} ) = E_{r,m}^{2} + E_{\theta ,m}^{2} + E_{\phi ,m}^{2}$$

5 $$I_{c} (r,\theta ,\phi ,\lambda_{p} ) = E_{r,c}^{2} + E_{\theta ,c}^{2} + E_{\phi ,c}^{2}$$

6 $$I_{sh} (r,\theta ,\phi ,\lambda_{p} ) = E_{r,sh}^{2} + E_{\theta ,sh}^{2} + E_{\phi ,sh}^{2}$$

here (*r*,*θ*,*ϕ*) are the spherical polar coordinates. *λ*_1_, *λ*_2_, *λ*_*3*_, …., *λ*_*k*_ represent different wavelengths present in the polychromatic light. *E*_*r,m*_, *E*_*r,c*_, and *E*_*r,sh*_ are the enhancements in the radial component of the electric field in the medium, core, and shell, respectively. *E*_*θ,m*_, *E*_*θ,c*_, and *E*_*θ,sh*_ are the enhancements in the angular component of the electric field in the medium, core, and shell, respectively. *E*_*ϕ,m*_, *E*_*ϕ,c*_, and *E*_*ϕ,sh*_ are the enhancements in the azimuthal component of the electric field in the medium, core, and shell, respectively. These components can be estimated using the following expressions.

7 $$E_{r,m} = E_{p} \left( {\lambda_{p} } \right)\cos \phi \mathop \sum \limits_{n = 1}^{\infty } C_{n} g_{n} a_{n}^{m} \left( {\lambda_{p} } \right)\left[ {\xi_{n}^{\prime \prime } \left( {k_{m} \left( {\lambda_{p} } \right)r} \right) + \xi_{n} \left( {k_{m} \left( {\lambda_{p} } \right)r} \right)} \right]P_{n}^{1} \left( {\cos \theta } \right)$$

8 $$E_{\theta ,m} = \frac{{E_{p} \left( {\lambda_{p} } \right)}}{{k_{m} \left( {\lambda_{p} } \right)r}}\cos \phi \mathop \sum \limits_{n = 1}^{\infty } C_{n} g_{n} \left[ {a_{n}^{m} \left( {\lambda_{p} } \right)\xi_{n}^{\prime } \left( {k_{m} \left( {\lambda_{p} } \right)r} \right)\tau_{n} \left( {\cos \theta } \right) - ib_{n}^{m} \left( {\lambda_{p} } \right)\xi_{n} \left( {k_{m} \left( {\lambda_{p} } \right)r} \right)\pi_{n} \left( {\cos \theta } \right)} \right]$$

9 $$E_{\phi ,m} = - \frac{{E_{p} \left( {\lambda_{p} } \right)}}{{k_{m} \left( {\lambda_{p} } \right)r}}\sin \phi \mathop \sum \limits_{n = 1}^{\infty } C_{n} g_{n} \left[ {a_{n}^{m} \left( {\lambda_{p} } \right)\xi_{n}^{\prime } \left( {k_{m} \left( {\lambda_{p} } \right)r} \right)\pi_{n} \left( {\cos \theta } \right) - ib_{n}^{m} \left( {\lambda_{p} } \right)\xi_{n} \left( {k_{m} \left( {\lambda_{p} } \right)r} \right)\tau_{n} \left( {\cos \theta } \right)} \right]$$

10 $$E_{r,sh} = - E_{p} \left( {\lambda_{p} } \right)\cos \phi \mathop \sum \limits_{n = 1}^{\infty } C_{n} g_{n} \left\{ \begin{gathered} a_{n}^{sh\alpha } \left( {\lambda_{p} } \right)\left[ {\psi_{n}^{\prime \prime } \left( {k_{sh} \left( {\lambda_{p} } \right)r} \right) + \psi_{n} \left( {k_{sh} \left( {\lambda_{p} } \right)r} \right)} \right] \hfill \\ - a_{n}^{sh\beta } \left( {\lambda_{p} } \right)\left[ {\xi_{n}^{\prime \prime } \left( {k_{sh} \left( {\lambda_{p} } \right)r} \right) + \xi_{n} \left( {k_{sh} \left( {\lambda_{p} } \right)r} \right)} \right] \hfill \\ \end{gathered} \right\}P_{n}^{1} \left( {\cos \theta } \right)$$

11 $$E_{\theta ,sh} = - \frac{{E_{p} \left( {\lambda_{p} } \right)}}{{k_{sh} \left( {\lambda_{p} } \right)r}}\cos \phi \mathop \sum \limits_{n = 1}^{\infty } C_{n} g_{n} \left\{ \begin{gathered} \left[ {a_{n}^{sh\alpha } \left( {\lambda_{p} } \right)\psi_{n}^{\prime } \left( {k_{sh} \left( {\lambda_{p} } \right)r} \right) - a_{n}^{sh\beta } \left( {\lambda_{p} } \right)\xi_{n}^{\prime } \left( {k_{sh} \left( {\lambda_{p} } \right)r} \right)} \right]\tau_{n} \left( {\cos \theta } \right) \hfill \\ - i\left[ {b_{n}^{sh\alpha } \left( {\lambda_{p} } \right)\psi_{n} \left( {k_{sh} \left( {\lambda_{p} } \right)r} \right) - b_{n}^{sh\beta } \left( {\lambda_{p} } \right)\xi_{n} \left( {k_{sh} \left( {\lambda_{p} } \right)r} \right)} \right]\pi_{n} \left( {\cos \theta } \right) \hfill \\ \end{gathered} \right\}$$

12 $$E_{\phi ,sh} = \frac{{E_{p} \left( {\lambda_{p} } \right)}}{{k_{sh} \left( {\lambda_{p} } \right)r}}\sin \phi \mathop \sum \limits_{n = 1}^{\infty } C_{n} g_{n} \left\{ \begin{gathered} \left[ {a_{n}^{sh\alpha } \left( {\lambda_{p} } \right)\psi_{n} \left( {k_{sh} \left( {\lambda_{p} } \right)r} \right) - a_{n}^{sh\beta } \left( {\lambda_{p} } \right)\xi_{n} \left( {k_{sh} \left( {\lambda_{p} } \right)r} \right)} \right]\pi_{n} \left( {\cos \theta } \right) \hfill \\ - i\left[ {b_{n}^{sh\alpha } \left( {\lambda_{p} } \right)\psi_{n}^{\prime } \left( {k_{sh} \left( {\lambda_{p} } \right)r} \right) - b_{n}^{sh\beta } \left( {\lambda_{p} } \right)\xi_{n}^{\prime } \left( {k_{sh} \left( {\lambda_{p} } \right)r} \right)} \right]\tau_{n} \left( {\cos \theta } \right) \hfill \\ \end{gathered} \right\}$$

13 $$E_{r,c} = - E_{p} \left( {\lambda_{p} } \right)\cos \phi \mathop \sum \limits_{n = 1}^{\infty } C_{n} g_{n} a_{n}^{c} \left( {\lambda_{p} } \right)\left[ {\psi_{n}^{\prime \prime } \left( {k_{c} \left( {\lambda_{p} } \right)r} \right) + \psi_{n} \left( {k_{c} \left( {\lambda_{p} } \right)r} \right)} \right]P_{n}^{1} \left( {\cos \theta } \right)$$

14 $$E_{\theta ,c} = - \frac{{E_{p} \left( {\lambda_{p} } \right)}}{{k_{c} \left( {\lambda_{p} } \right)r}}\cos \phi \mathop \sum \limits_{n = 1}^{\infty } C_{n} g_{n} \left[ {a_{n}^{c} \left( {\lambda_{p} } \right)\psi_{n}^{\prime } \left( {k_{c} \left( {\lambda_{p} } \right)r} \right)\tau_{n} \left( {\cos \theta } \right) - ib_{n}^{c} \left( {\lambda_{p} } \right)\psi_{n} \left( {k_{c} \left( {\lambda_{p} } \right)r} \right)\pi_{n} \left( {\cos \theta } \right)} \right]$$

15 $$E_{\phi ,c} = \frac{{E_{p} \left( {\lambda_{p} } \right)}}{{k_{c} \left( {\lambda_{p} } \right)r}}\sin \phi \mathop \sum \limits_{n = 1}^{\infty } C_{n} g_{n} \left[ {a_{n}^{c} \left( {\lambda_{p} } \right)\psi_{n}^{\prime } \left( {k_{c} \left( {\lambda_{p} } \right)r} \right)\pi_{n} \left( {\cos \theta } \right) - ib_{n}^{c} \left( {\lambda_{p} } \right)\psi_{n} \left( {k_{c} \left( {\lambda_{p} } \right)r} \right)\tau_{n} \left( {\cos \theta } \right)} \right]$$

16 $$C_{n} {\text{ = i}}^{{\left( {n + 1} \right)}} \left( { - 1} \right)^{n} \frac{2n + 1}{{n\left( {n + 1} \right)}}$$

17 $$a_{n}^{m} \left( {\lambda_{p} } \right) = X_{3} /X_{1} ;a_{n}^{c} \left( {\lambda_{p} } \right) = X_{4} /X_{1} ;a_{n}^{sh\alpha } \left( {\lambda_{p} } \right) = X_{5} /X_{1} ;a_{n}^{sh\beta } \left( {\lambda_{p} } \right) = X_{6} /X_{1}$$

18 $$b_{n}^{m} \left( {\lambda_{p} } \right) = X_{7} /X_{2} ;b_{n}^{c} \left( {\lambda_{p} } \right) = X_{4} /X_{2} ;b_{n}^{sh\alpha } \left( {\lambda_{p} } \right) = X_{8} /X_{2} ;b_{n}^{sh\beta } \left( {\lambda_{p} } \right) = X_{9} /X_{2}$$

19 $$\begin{aligned} X_{1} & = n_{sh}^{2} \xi^{\prime}\left( {k_{m} \left( {\lambda_{p} } \right)R_{sh} } \right)\psi^{\prime } \left( {k_{c} \left( {\lambda_{p} } \right)R_{c} } \right)M_{1} + n_{m}^{{}} n_{c}^{{}} \xi \left( {k_{m} \left( {\lambda_{p} } \right)R_{sh} } \right)\psi \left( {k_{c} \left( {\lambda_{p} } \right)R_{c} } \right)M_{2} \\ & \quad + n_{sh}^{{}} n_{c}^{{}} \xi^{\prime}\left( {k_{m} \left( {\lambda_{p} } \right)R_{sh} } \right)\psi \left( {k_{c} \left( {\lambda_{p} } \right)R_{c} } \right)M_{3} + n_{m}^{{}} n_{sh}^{{}} \xi \left( {k_{m} \left( {\lambda_{p} } \right)R_{sh} } \right)\psi^{\prime } \left( {k_{c} \left( {\lambda_{p} } \right)R_{c} } \right)M_{4} \\ \end{aligned}$$

20 $$\begin{aligned} X_{2} & = n_{m} n_{c} \xi^{^{\prime}} \left( {k_{m} (\lambda_{p} )R_{sh} } \right)\psi^{^{\prime}} \left( {k_{c} (\lambda_{p} )R_{c} } \right)M_{1} + n_{sh}^{2} \xi \left( {k_{m} (\lambda_{p} )R_{sh} } \right)\psi \left( {k_{c} (\lambda_{p} )R_{c} } \right)M_{2} \\ & \quad + n_{m} n_{sh} \xi^{^{\prime}} \left( {k_{m} (\lambda_{p} )R_{sh} } \right)\psi \left( {k_{c} (\lambda_{p} )R_{c} } \right)M_{3} + n_{sh} n_{c} \xi (k_{m} (\lambda_{p} )R_{sh} )\psi^{^{\prime}} \left( {k_{c} (\lambda_{p} )R_{c} } \right)M_{4} \\ \end{aligned}$$

21 $$\begin{aligned} X_{3} & = n_{sh}^{2} \psi^{\prime } \left( {k_{c} \left( {\lambda_{p} } \right)R_{c} } \right)\psi^{\prime}\left( {k_{m} \left( {\lambda_{p} } \right)R_{sh} } \right)M_{1} + n_{c} n_{m} \psi \left( {k_{c} \left( {\lambda_{p} } \right)R_{c} } \right)\psi \left( {k_{m} \left( {\lambda_{p} } \right)R_{sh} } \right)M_{2} \\ & \quad + n_{c} n_{sh} \psi \left( {k_{c} \left( {\lambda_{p} } \right)R_{c} } \right)\psi^{\prime } \left( {k_{m} \left( {\lambda_{p} } \right)R_{sh} } \right)M_{3} + n_{m} n_{sh} \psi^{\prime } \left( {k_{c} \left( {\lambda_{p} } \right)R_{c} } \right)\psi \left( {k_{m} \left( {\lambda_{p} } \right)R_{sh} } \right)M_{4} \\ \end{aligned}$$

22 $$X_{4} = n_{c}^{{}} n_{sh}^{{}} (M_{5} - M_{6} )\left[ {\xi \left( {k_{sh} \left( {\lambda_{p} } \right)R_{c} } \right)\psi^{\prime } \left( {k_{sh} \left( {\lambda_{p} } \right)R_{c} } \right) - \xi^{\prime}\left( {k_{sh} \left( {\lambda_{p} } \right)R_{c} } \right)\psi \left( {k_{sh} \left( {\lambda_{p} } \right)R_{c} } \right)} \right]$$

23 $$X_{5} = - n_{sh} \left\{ \begin{gathered} n_{sh}^{{}} \psi^{\prime } \left( {k_{c} \left( {\lambda_{p} } \right)R_{c} } \right)\left[ {\xi \left( {k_{sh} \left( {\lambda_{p} } \right)R_{c} } \right)M_{5} - \xi \left( {k_{sh} \left( {\lambda_{p} } \right)R_{c} } \right)M_{6} } \right] \hfill \\ + n_{c}^{{}} \psi \left( {k_{c} \left( {\lambda_{p} } \right)R_{c} } \right)\left[ {\xi^{\prime } \left( {k_{sh} \left( {\lambda_{p} } \right)R_{c} } \right)M_{6} - \xi^{\prime}\left( {k_{sh} \left( {\lambda_{p} } \right)R_{c} } \right)M_{5} } \right] \hfill \\ \end{gathered} \right\}$$

24 $$X_{6} = - n_{sh} \left\{ \begin{gathered} n_{sh}^{{}} \psi^{\prime } \left( {k_{c} \left( {\lambda_{p} } \right)R_{c} } \right)\left[ {\psi \left( {k_{sh} \left( {\lambda_{p} } \right)R_{c} } \right)M_{5} - \psi \left( {k_{sh} \left( {\lambda_{p} } \right)R_{c} } \right)M_{6} } \right] \hfill \\ + n_{c}^{{}} \psi \left( {k_{c} \left( {\lambda_{p} } \right)R_{c} } \right)\left[ {\psi^{\prime } \left( {k_{sh} \left( {\lambda_{p} } \right)R_{c} } \right)M_{6} - \psi^{\prime}\left( {k_{sh} \left( {\lambda_{p} } \right)R_{c} } \right)M_{5} } \right] \hfill \\ \end{gathered} \right\}$$

25 $$\begin{aligned} X_{7} & = n_{c}^{{}} n_{m} \psi^{\prime } \left( {k_{c} \left( {\lambda_{p} } \right)R_{c} } \right)\psi^{\prime}\left( {k_{m} \left( {\lambda_{p} } \right)R_{sh} } \right)M_{1} + n_{sh} n_{m} \psi \left( {k_{c} \left( {\lambda_{p} } \right)R_{c} } \right)\psi \left( {k_{m} \left( {\lambda_{p} } \right)R_{sh} } \right)M_{2} \\ & \quad + n_{sh}^{2} \psi \left( {k_{c} \left( {\lambda_{p} } \right)R_{c} } \right)\psi^{\prime } \left( {k_{m} \left( {\lambda_{p} } \right)R_{sh} } \right)M_{3} + n_{c} n_{sh} \psi^{\prime } \left( {k_{c} \left( {\lambda_{p} } \right)R_{c} } \right)\psi \left( {k_{m} \left( {\lambda_{p} } \right)R_{sh} } \right)M_{4} \\ \end{aligned}$$

26 $$X_{8} = - n_{sh} \left\{ \begin{gathered} n_{c}^{{}} \psi^{\prime } \left( {k_{c} \left( {\lambda_{p} } \right)R_{c} } \right)\left[ {\xi \left( {k_{sh} \left( {\lambda_{p} } \right)R_{c} } \right)M_{5} - \xi \left( {k_{sh} \left( {\lambda_{p} } \right)R_{c} } \right)M_{6} } \right] \hfill \\ + n_{sh}^{{}} \psi \left( {k_{c} \left( {\lambda_{p} } \right)R_{c} } \right)\left[ {\xi^{\prime } \left( {k_{sh} \left( {\lambda_{p} } \right)R_{c} } \right)M_{6} - \xi^{\prime}\left( {k_{sh} \left( {\lambda_{p} } \right)R_{c} } \right)M_{5} } \right] \hfill \\ \end{gathered} \right\}$$

27 $$X_{9} = - n_{sh} \left\{ \begin{gathered} n_{c}^{{}} \psi^{\prime } \left( {k_{c} \left( {\lambda_{p} } \right)R_{c} } \right)\left[ {\psi \left( {k_{sh} \left( {\lambda_{p} } \right)R_{c} } \right)M_{5} - \psi \left( {k_{sh} \left( {\lambda_{p} } \right)R_{c} } \right)M_{6} } \right] \hfill \\ + n_{sh}^{{}} \psi \left( {k_{c} \left( {\lambda_{p} } \right)R_{c} } \right)\left[ {\psi^{\prime } \left( {k_{sh} \left( {\lambda_{p} } \right)R_{c} } \right)M_{6} - \psi^{\prime}\left( {k_{sh} \left( {\lambda_{p} } \right)R_{c} } \right)M_{5} } \right] \hfill \\ \end{gathered} \right\}$$

28 $$\begin{gathered} M_{1} = \xi \left( {k_{sh} \left( {\lambda_{p} } \right)R_{c} } \right)\psi \left( {k_{sh} \left( {\lambda_{p} } \right)R_{sh} } \right) - \xi \left( {k_{sh} \left( {\lambda_{p} } \right)R_{sh} } \right)\psi \left( {k_{sh} \left( {\lambda_{p} } \right)R_{c} } \right); \hfill \\ M_{2} = \xi^{\prime}\left( {k_{sh} \left( {\lambda_{p} } \right)R_{c} } \right)\psi^{\prime } \left( {k_{sh} \left( {\lambda_{p} } \right)R_{sh} } \right) - \xi^{\prime}\left( {k_{sh} \left( {\lambda_{p} } \right)R_{sh} } \right)\psi^{\prime } \left( {k_{sh} \left( {\lambda_{p} } \right)R_{c} } \right) \hfill \\ \end{gathered}$$

29 $$\begin{gathered} M_{3} = \xi \left( {k_{sh} \left( {\lambda_{p} } \right)R_{sh} } \right)\psi^{\prime } \left( {k_{sh} \left( {\lambda_{p} } \right)R_{c} } \right) - \xi^{\prime}\left( {k_{sh} \left( {\lambda_{p} } \right)R_{c} } \right)\psi \left( {k_{sh} \left( {\lambda_{p} } \right)R_{sh} } \right); \hfill \\ M_{4} = \xi^{\prime}\left( {k_{sh} \left( {\lambda_{p} } \right)R_{sh} } \right)\psi \left( {k_{sh} \left( {\lambda_{p} } \right)R_{c} } \right) - \xi \left( {k_{sh} \left( {\lambda_{p} } \right)R_{c} } \right)\psi^{\prime } \left( {k_{sh} \left( {\lambda_{p} } \right)R_{sh} } \right) \hfill \\ \end{gathered}$$

30 $$M_{5} = \xi \left( {k_{m} \left( {\lambda_{p} } \right)R_{sh} } \right)\psi^{\prime } \left( {k_{m} \left( {\lambda_{p} } \right)R_{sh} } \right);M_{6} = \xi^{\prime}\left( {k_{m} \left( {\lambda_{p} } \right)R_{sh} } \right)\psi \left( {k_{m} \left( {\lambda_{p} } \right)R_{sh} } \right)$$

where *R*_*c*_ and *R*_*sh*_ are the radii of the core and shell, respectively. *k*_*m*_, *k*_*sh*_, and *k*_*c*_ are the propagation constants of light in the surrounding medium, shell, and core, respectively. *ξ*_*n*_ and *ψ*_*n*_ are the Riccati-Hankel and Bessel functions, respectively^65^. *τ*_*n*_ and *π*_*n*_ are the angle-dependent associated Legendre polynomials, respectively. *g*_*n*_ is the correcting Bromwich coefficient, which is generally expressed as^21^

31 $$g_{n} = \frac{2n + 1}{{\pi n\left( {n + 1} \right)}}\frac{1}{{\left( { - 1} \right)^{n} i^{n} }}\int\limits_{0}^{\pi } {\int\limits_{0}^{\infty } {ik_{m} r\sin^{2} \theta \times f \times \exp \left( { - ik_{m} r\cos \theta } \right)\psi {}_{n}^{1} \left( {k_{m} r} \right)P_{n}^{1} \left( {\cos \theta } \right)d\theta d\left( {k_{m} r} \right)} }$$

All parameters and functions in this expression are explained in Ref.^21^. Further, Gouesbet et al. simplified Eq. (31) as follows^66^

32 $$g_{n} = e^{{ - {{\rho_{n}^{2} } \mathord{\left/ {\vphantom {{\rho_{n}^{2} } {\omega_{0}^{2} }}} \right. \kern-\nulldelimiterspace} {\omega_{0}^{2} }}}}$$

here *ρ*_*n*_ = (*n* + 0.5)(*λ*/2π). It is to be noted that the Eq. (32) will be valid only when the center of the microsphere is located at the center of the beam waist (as in the present study).

The expression for *E*_*p*_(*λ*_*p*_) is given below.

33 $$E_{p} (\lambda_{p} ) = \frac{{E_{0} (\lambda_{p} )}}{{\mathop \Sigma \limits_{p = 1}^{k} E_{0} (\lambda_{p} )}};\quad \mathop \Sigma \limits_{p = 1}^{k} E_{p} (\lambda_{p} ) = 1$$

here *E*_0_(*λ*_*p*_) is the electric field amplitude of the radiation of wavelength *λ*_*p*_.

It is important to note that all these equations are dependent on the refractive index (*n*) of the considered material. It is well known that the *n* value depends upon *λ*_*p*_. In the case of polychromatic illumination, one has to consider the dispersion of the refractive index of the material. In the present study, polystyrene (PS) and fused silica have been considered as particle materials. The dispersion relations for PS^67^ and fused silica^68^ are given below in the same order.

34 $$n^{2} - 1 = \frac{{1.4435\lambda_{p}^{2} }}{{\lambda_{p}^{2} - 0.020216}}$$

35 $$n^{2} - 1 = \frac{{0.6961663\lambda_{p}^{2} }}{{\lambda_{p}^{2} - 0.0684043^{2} }} + \frac{{0.4079426\lambda_{p}^{2} }}{{\lambda_{p}^{2} - 0.1162414^{2} }} + \frac{{0.8974794\lambda_{p}^{2} }}{{\lambda_{p}^{2} - 9.896161^{2} }}$$

**Figure 2:**
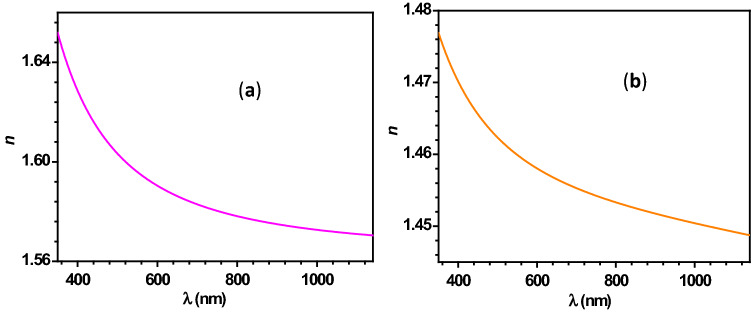
Panels (**a**,**b**) represent the dispersion of the refractive index of PS and fused silica, respectively.

In addition, the dispersion curves for PS and fused silica are plotted using Eqs. (34 and 35) and shown in Fig. 2.

## Results and discussion

### Emission spectra of different polychromatic light sources

As mentioned in the introduction, the main aim of the present work is to understand the characteristic parameters of PNJs of single solid/core–shell microspheres under focused polychromatic illumination. So, to perform the theoretical investigation on PNJs of single dielectric microspheres using equations shown in the above section, the number of wavelengths present in the incident polychromatic light and electric field amplitude at each wavelength are necessary. These details can be obtained easily by recording the spectrum of polychromatic light. Therefore, before starting the theoretical investigation, the emission spectra of different polychromatic light sources such as Halogen lamp, white LED, SC source, and Hg arc lamp available in our laboratory are recorded using the charge-coupled device (CCD) spectrometer, and obtained spectra are used in the present study.

**Figure 3:**
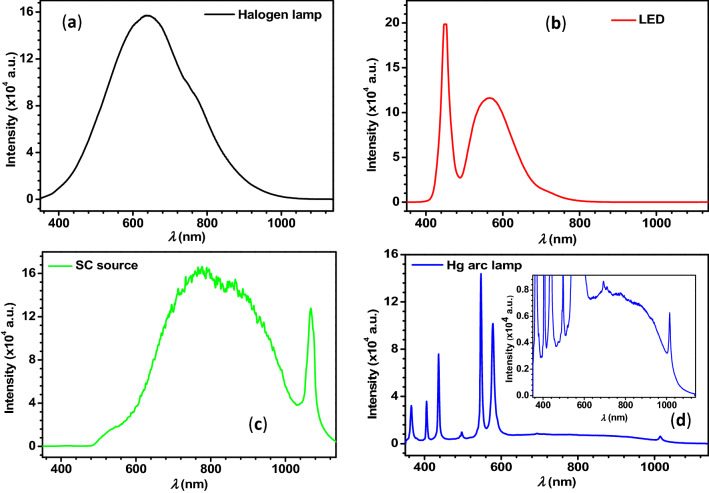
Panels (**a**–**d**) represent the spectra of Halogen lamp, white LED, SC source, and Hg arc lamp, respectively. Inset in panel (**d**) represents a portion of the vertically zoomed spectrum.

From Panel (a) of Fig. 3, it is clear that the spectrum of the Halogen lamp is continuous and spanned from 380 to 1000 nm. The peak maximum is observed at 640 nm. In the case of white LED, two peaks are observed. The central wavelengths of sharp and broad peaks are observed at 450 nm and 565 nm, respectively. In the case of SC source operated at lower power, the spectrum is started from 500 to 1100 nm. The supercontinuum radiation is generated from a photonic crystal fiber and a sharp peak in the spectrum at 1064 nm corresponds to the pump laser (i.e., picosecond diode laser). The background in the spectrum of the Hg arc lamp is spanned from 400 to 1100 nm. As expected, several sharp peaks are observed in the range from 380 to 600 nm.

### PNJs generated by single PS microspheres kept in water medium

**Figure 4:**
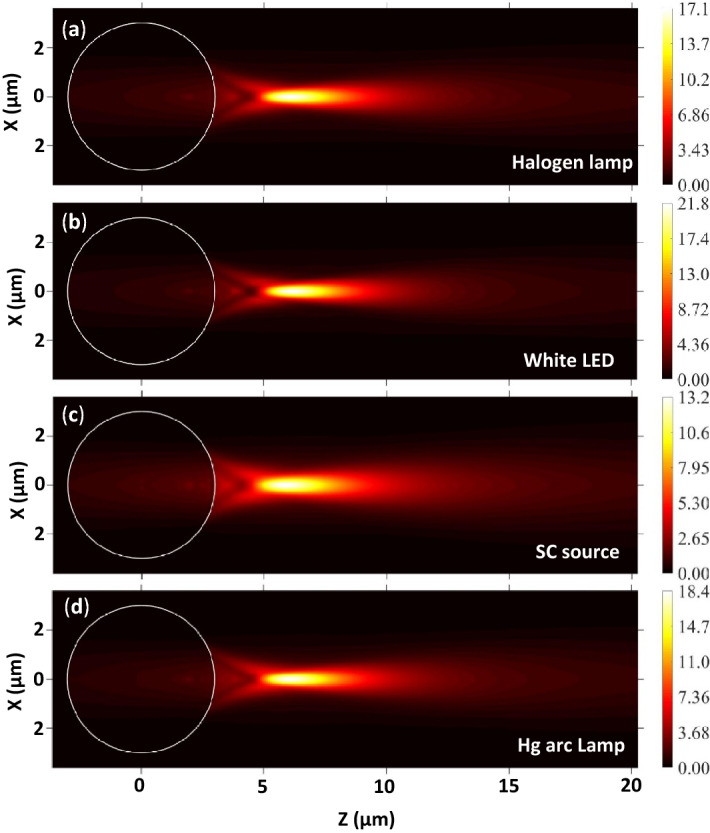
EFIE distribution inside and outside a PS microsphere illuminated by polychromatic light from different sources. Here *R*_*s*_ = 3 µm, *ω*_*0*_ = 3 µm, and *n*_*m*_ = 1.333. The wavelength-dependent refractive indices of PS are estimated from Eq. (34) and used for these simulations.

Using the spectra of different polychromatic light sources (Fig. 3) and theoretical equations mentioned in Sect. 2, the EFIE distribution inside and outside single PS microspheres is plotted and shown in Fig. 4.

In all panels, a white circle represents the microsphere boundary. The blue and red colors represent the lower and higher EFIE values. From this figure, it can be observed that the maximum EFIE (*η*_*max*_) values obtained for all the mentioned light sources are not the same due to the difference in the wavelengths and electric field amplitude of the incident radiation. The PNJ generated through the microsphere due to the SC source illumination is the least convergent among all shown in the figure because the shorter wavelengths are not present in the spectrum. In addition, the radiation from the SC source contains several larger wavelengths (beyond 1000 nm). Since the spectrum of the LED contains one intense peak at a lower wavelength region, the *η*_*max*_ value observed for this source is slightly larger as compared to those obtained for other light sources.

**Figure 5:**
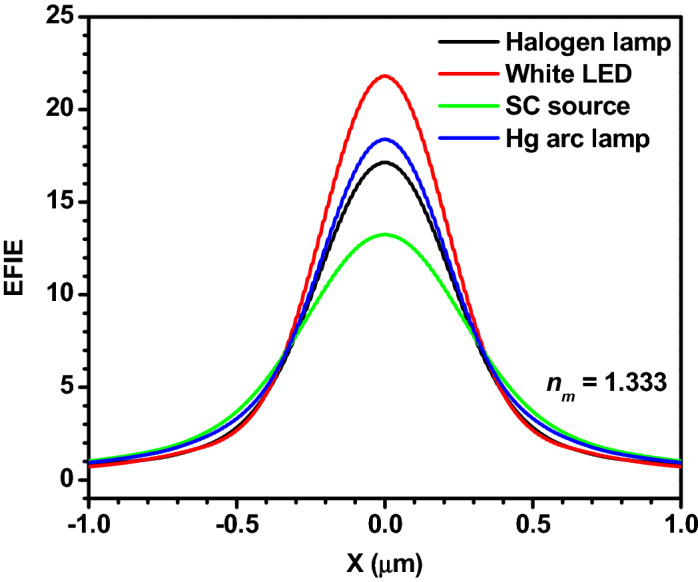
Variation in the EFIE along the transversal axes of the PNJs generated by a PS microsphere illuminated by polychromatic light from different sources. Here *R*_*s*_ = 3 µm, *ω*_*0*_ = 3 µm, and *n*_*m*_ = 1.333.

Another characteristic parameter that plays a major role in several applications is the width of the PNJ. Therefore, the line scans along the transversal axis are taken across the PNJs generated using different light sources and shown in Fig. 5. In this figure, X = 0 indicates the center of the PNJs where the EFIE is maximum. The effective width of the PNJs (*W*_*eff*_) is nothing but the width of the peaks. Here the *W*_*eff*_ is found smaller for the case of LED and it is larger for the SC source. It is worth mentioning here that the *W*_*eff*_ values are reasonably larger due to the smaller relative refractive index of the PS microspheres in the water medium. Therefore, these PNJs are not useful for some applications such as PNJ assisted super-resolution white light nanoscopy and photoacoustic spectroscopy. However, the *W*_*eff*_ can be lowered significantly in water medium if we use microspheres that have higher refractive indices.

**Figure 6:**
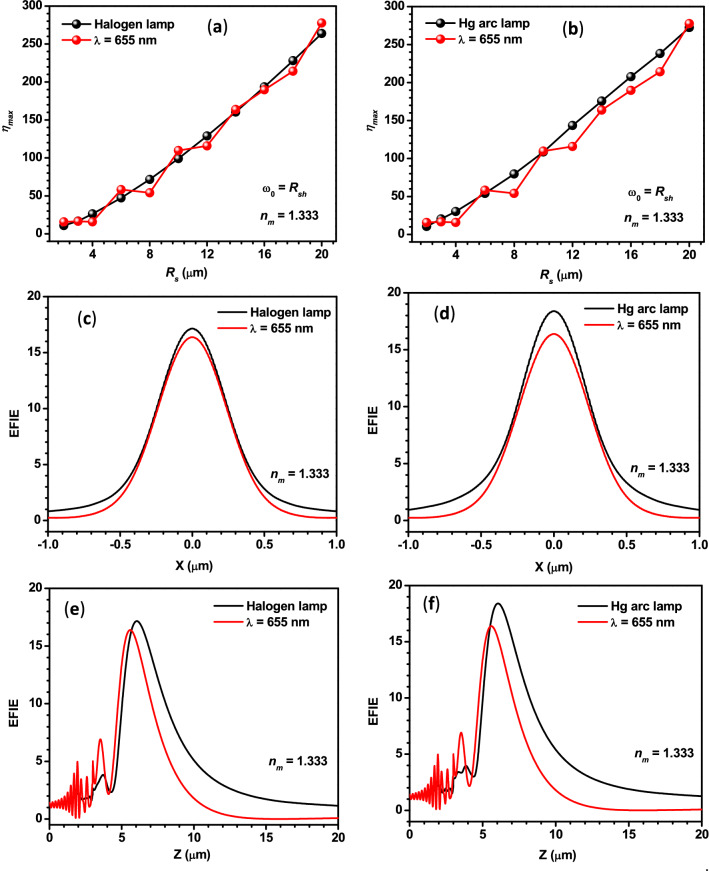
Panels (**a**,**b**) show the comparison between the *η*_*max*_ values obtained for monochromatic light of *λ* = 655 nm and polychromatic light from two different sources. Panels (**c**,**d**) show the comparison between the variation in the EFIE along the transversal axes of the PNJs generated by a PS microsphere illuminated by monochromatic light *λ* = 655 nm and polychromatic light from different sources. Panels (**e**,**f**) show the same along the longitudinal axes. In the case of the last four panels, *R*_*s*_ = 3 µm, and *ω*_*0*_ = 3 µm.

As mentioned in the introduction, several researchers have numerically/theoretically studied the PNJs generated by single dielectric microparticles under monochromatic illumination. It has been reported that the characteristic parameters of the PNJs strongly depend upon the wavelength of the incident light, size, shape, and relative refractive index of the microparticles. In addition, there is always a little deviation between the results obtained using different commercial software and theoretical approaches. Therefore, to have a proper comparison between the PNJs obtained by monochromatic and polychromatic illumination, we have performed the theoretical investigation on PNJ of single microspheres (*R*_*s*_ = 3 µm) illuminated by monochromatic light of different wavelengths and found that the *η*_*max*_ values obtained with monochromatic light of *λ* = 655 nm, Halogen lamp, and Hg arc lamp are quite close. To find out whether this is the same even for microspheres of different sizes or not, we have also estimated the *η*_*max*_ values for the microspheres of different sizes, illuminated by monochromatic light of *λ* = 655 nm, Halogen lamp, and Hg arc lamp and the obtained values are shown in panels (a) and (b) of Fig. 6, separately. In both the panels, overall, the *η*_*max*_ value is found to increase with the *R*_*s*_ value. This could be due to the predominant forward Mie scattering in the case of larger microspheres. It is to be noted that fluctuations in the curve for *λ* = 655 nm indicate the partial excitation of optical resonances or whispering gallery modes of the microspheres of specific sizes. From both the panels, we can conclude that the *η*_*max*_ values obtained for the Halogen light, Hg arc lamp, and monochromatic light of *λ* = 655 nm are nearby for all *R*_*s*_ values.

For comparing the *W*_*eff*_ values, the line scans along the transversal axes of the PNJs of the PS microsphere (*R*_*s*_ = 3 µm) illuminated by monochromatic light of *λ* = 655 nm, polychromatic light from Halogen lamp and Hg arc lamp are plotted and shown in panels (c) and (d) of Fig. 6. From these Panels, we can easily conclude that the *W*_*eff*_ values are also nearly the same.

For comparing the lengths of the PNJs, the line scans along the optical axes of the PNJs of the PS microsphere (*R*_*s*_ = 3 µm) illuminated by monochromatic light of *λ* = 655 nm, polychromatic light from Halogen lamp and Hg arc lamp are plotted and shown in panels (e) and (f) of Fig. 6. It is to be noted that the FWHM of the peaks shown in these two panels represents the effective length of the PNJs (*L*_*eff*_). From these figures, it is clear that the *L*_*eff*_ is slightly more in the case of both polychromatic sources.

### PNJs generated by single PS microspheres kept in air medium

**Figure 7:**
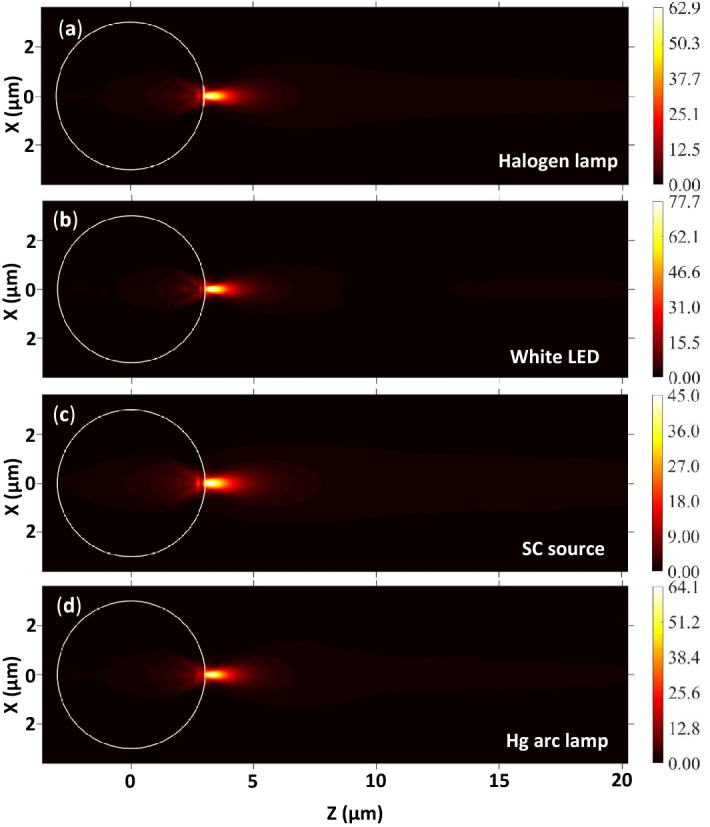
EFIE distribution inside and outside a PS microsphere illuminated by polychromatic light from different sources. Here *R*_*s*_ = 3 µm, *ω*_*0*_ = 3 µm, and *n*_*m*_ = 1.0.

Figure 7 shows the EFIE distribution inside and outside single PS microspheres (in air medium) illuminated by focused polychromatic light from different sources. As in the water medium, the *η*_*max*_ values obtained from the spectra of all polychromatic sources are slightly different. The *η*_*max*_ observed in the air medium is significantly larger as compared to those obtained in the water medium. This is due to the strong forward Mie scattering in the air medium due to the larger relative refractive index of the microsphere. Here also, (1) the *η*_*max*_ is larger in the case of white LED and it is smaller for the SC source, and (2) the width and length of the PNJs are significantly smaller in the air as compared to the water medium. Therefore, these PNJs could be recommended for PNJ assisted white light nanoscopy, photoacoustic spectroscopy, etc.

**Figure 8:**
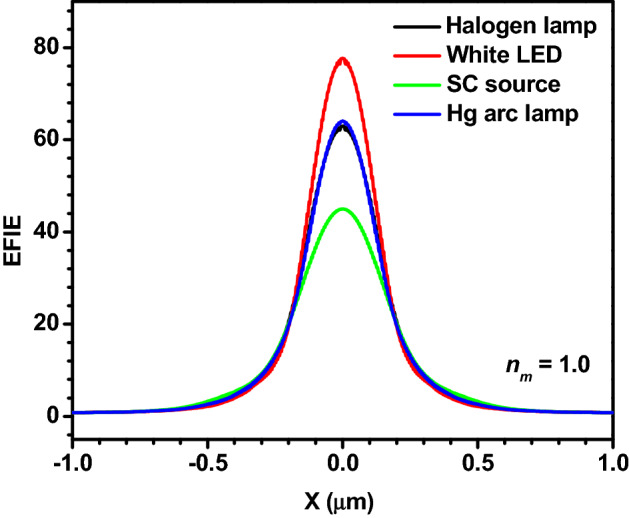
Variation in the EFIE along the transversal axes of the PNJ generated by a PS microsphere illuminated by polychromatic light from different sources. Here *R*_*s*_ = 3 µm, *ω*_*0*_ = 3 µm, and *n*_*m*_ = 1.0.

Figure 8 represents the line scans along the transversal axes of the PNJs shown in Fig. 7. In this figure also, X = 0 indicates the center of the PNJs where the EFIE is maximum. The width of the PNJs is nothing but the width of the peaks. It is apparent that only one peak is observed at X = 0 for all light sources but the side lobes are completely disappeared irrespective of the polychromatic light source. However, in the case of monochromatic illumination, along with the main peak at X = 0, several side lobes are observed in the air medium. The number and amplitude of the side lobes are significantly decreased when the *λ* value is increased from 400 to 1000 nm (results not shown). Here also, relatively, the width of the PNJ obtained for the SC source is slightly larger.

**Figure 9:**
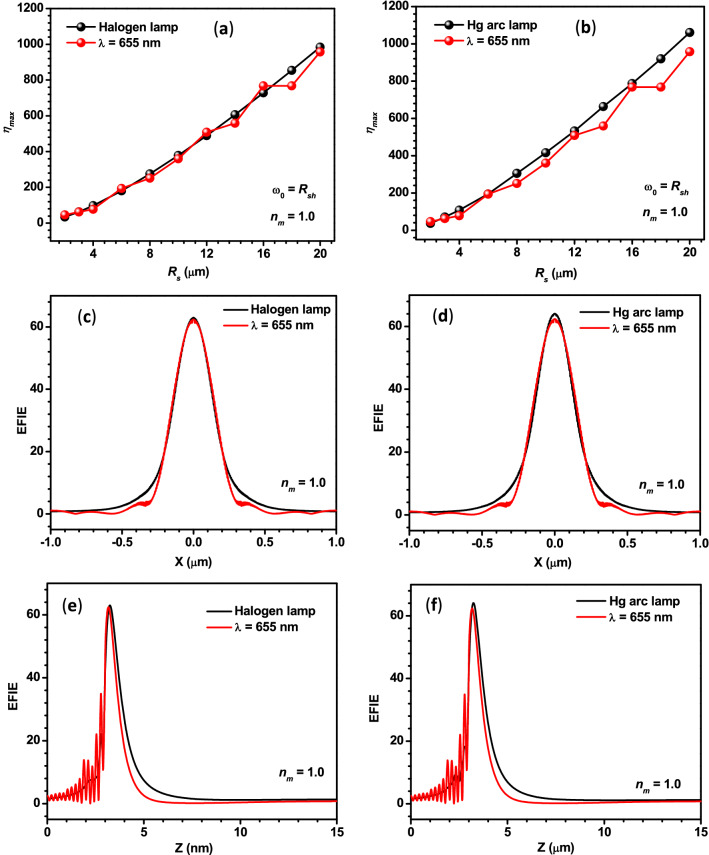
Panels (**a**,**b**) show the comparison between the *η*_*max*_ values obtained for monochromatic light of λ = 655 nm and polychromatic light from two different sources. Panels (**c**,**d**) show the comparison between the variation in the EFIE along the transversal axes of the PNJs generated by a PS microsphere illuminated by monochromatic light of λ = 655 nm and polychromatic light from different sources. Panels (**e**,**f**) show the same along the longitudinal axes. In the case of the last four panels, *R*_*s*_ = 3 µm, and *ω*_*0*_ = 3 µm.

In the air medium also, at first, the EFIE distribution inside and outside a PS microsphere (*R*_*s*_ = 3 µm) is plotted using monochromatic light of different *λ* values. From Panels (a) and (b) of Fig. 9, it is apparent that the *η*_*max*_ values of microspheres having different *R*_*s*_ obtained for monochromatic light of *λ* = 655 nm, polychromatic light from Halogen and Hg arc lamps are nearly the same. In addition, from Panels (c–f) of Fig. 9, it can be observed that the *W*_*eff*_ and *L*_*eff*_ values obtained for monochromatic light of *λ* = 655 nm, polychromatic light from Halogen and Hg arc lamps are close. Therefore, from Figs. (6 and 9), we can safely conclude that one can use the characteristic parameters of the PNJ obtained with monochromatic light of *λ* = 655 nm to interpret the experimental results obtained with the PNJs generated in air and water media, using the polychromatic light from Hg arc and Halogen lamps, which are the most commonly used in different types of microscopes. A similar observation is found for the silica microspheres as well (see “Supplementary information”). This observation is very useful for the researchers because generating PNJs numerically using monochromatic light is simple but the generation of PNJs numerically using polychromatic light is a difficult task and it demands a sophisticated computing facility.

A comparison between the EFIE distribution of the PNJs obtained from the PS microsphere under the illumination of polychromatic light from a white LED and SC source with the monochromatic light of different wavelengths is also made for finding the suitable wavelength of monochromatic radiation.

**Figure 10:**
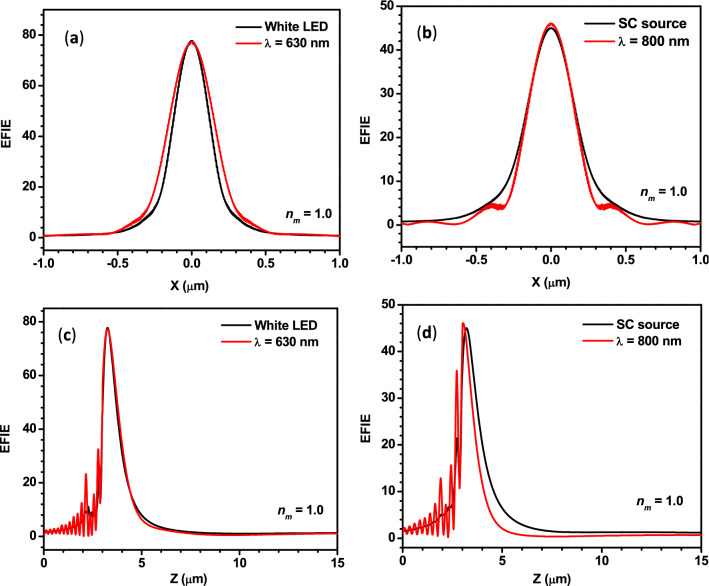
Panels (**a**,**b**) show the comparison between the variation in the EFIE along the transversal axes of the PNJs generated by a PS microsphere illuminated by monochromatic and polychromatic light from different sources. Panels (**c**,**d**) show the same along the longitudinal axes. For all the four panels, *R*_*s*_ = 3 µm and *ω*_*0*_ = 3 µm.

It can be observed from Panels (a) and (c) of Fig. 10 that the *W*_*eff*_ and *L*_*eff*_ values obtained for monochromatic light of *λ* = 630 nm and polychromatic light from LED are very close. Similar behavior can be observed from Panels (b) and (d) of Fig. 10 for monochromatic light of *λ* = 800 nm and polychromatic light from SC source. Thus, the characteristic parameters of the PNJs obtained from the illumination of the monochromatic light of the mentioned wavelengths can be used to interpret the experimental results for the PNJs under the illumination by respective polychromatic light sources as mentioned earlier in this section.

### Role of ***ω***_***0***_ on the PNJs of single microspheres under polychromatic illumination

**Figure 11:**
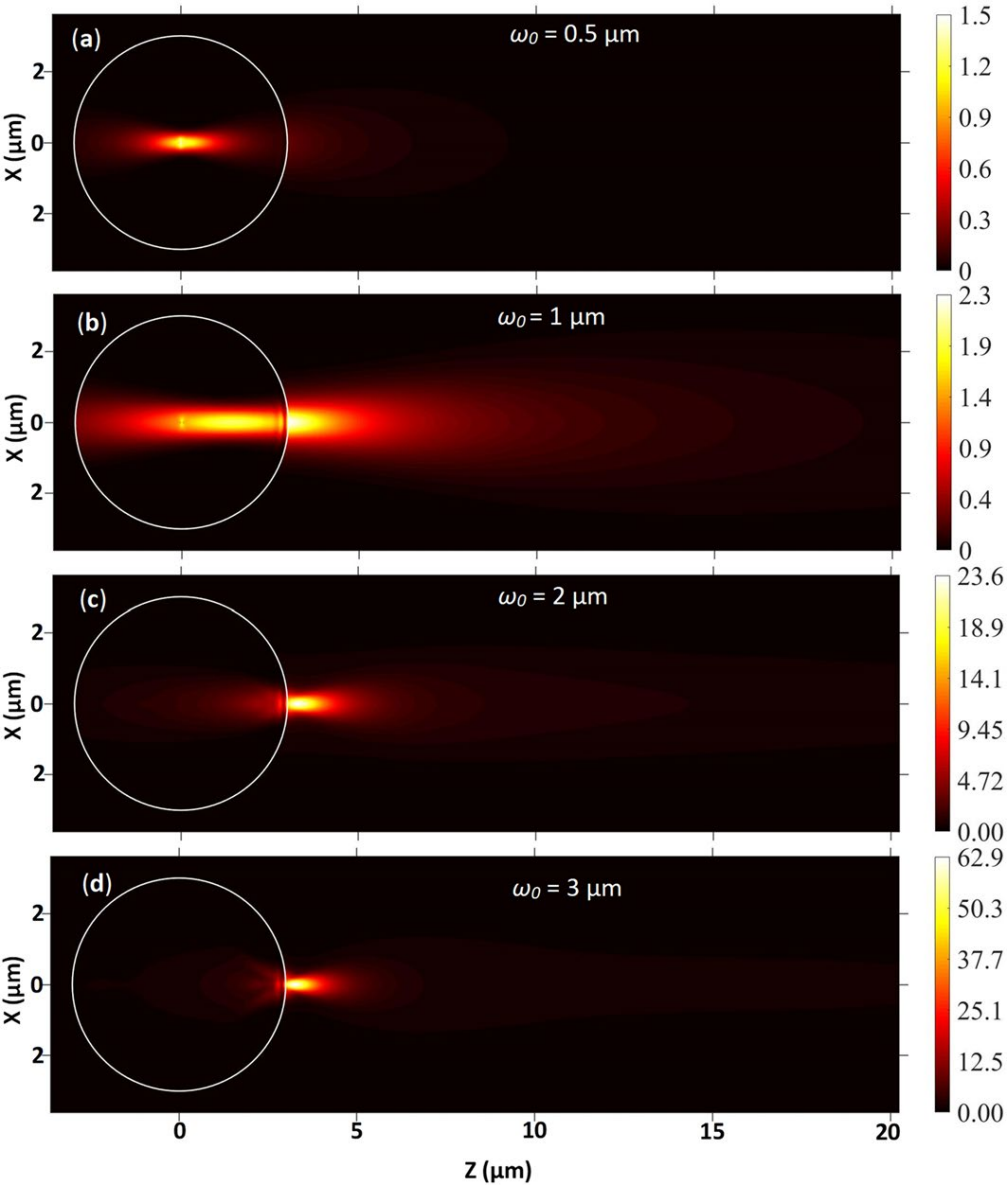
The role of *ω*_*0*_ on the EFIE inside and outside a PS microsphere illuminated by focused polychromatic light. Here *R*_*s*_ = 3 µm and *n*_*m*_ = 1.0. The spectrum of the Halogen lamp is used for generating all figures.

Experimentally, the PNJs are generated by focusing the polychromatic light on single microspheres with the help of a microscopic objective lens with different numerical aperture (NA) values. It is well known that the NA value directly affects the *ω*_*0*_. Though, the effect of *ω*_*0*_ on the EFIE of the PNJ due to monochromatic illumination is reported in the literature^69^, the same due to the polychromatic illumination is again not present in the literature. Therefore, to understand the effect of *ω*_*0*_ on the PNJs of single microspheres under polychromatic illumination theoretically, the EFIE distribution inside and outside single microspheres is plotted by varying the *ω*_*0*_ value. Figure 11 shows the EFIE distribution of a single PS microsphere of *R*_*s*_ = 3 µm obtained for different *ω*_*0*_ values. Here the emission spectrum of Halogen light shown in panel (a) of Fig. 3 is used for generating all panels in Fig. 11.

From Fig. 11, it can be observed that for a very small *ω*_*0*_ (= 0.5 µm) compared to the size of the microsphere, there is no formation of PNJ. The incident light is focused at the centre of the microsphere. When *ω*_*0*_ = 1 µm then the PNJ is partially visible outside the microsphere. But a reasonable amount of light is still confined inside the microsphere. Upon increasing the *ω*_*0*_ value from 1 to 2 µm, the PNJ is clearly visible outside the microsphere. However, the value of *η*_*max*_ is low due to the weak confinement of the PNJ. When *ω*_*0*_ = *R*_*s*_, the value of *η*_*max*_ is increased further which indicates the improvement in the confinement of PNJ. These results indicate that one has to use the focused polychromatic beam with *ω*_*0*_ = *R*_*s*_ to generate the tightly confined PNJs from single dielectric microspheres.

### PNJs generated by silica core-PS shell microspheres kept in air medium

**Figure 12:**
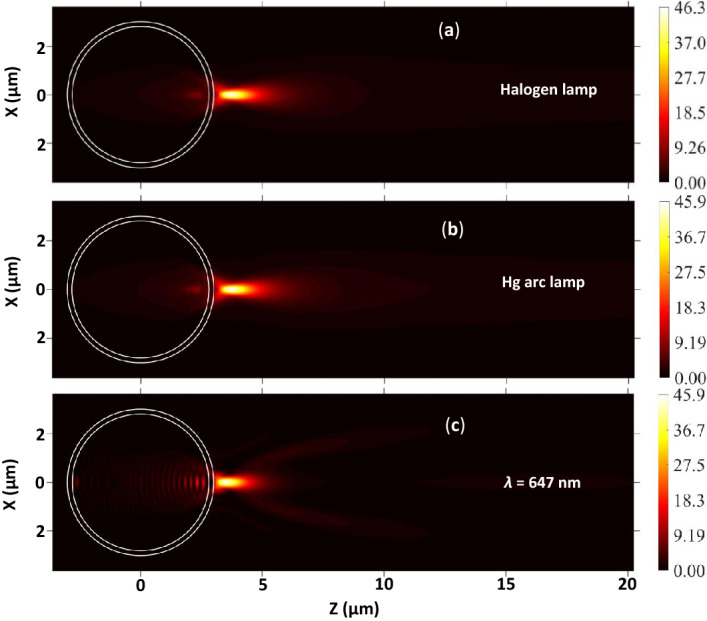
(**a**–**c**) represent the EFIE distribution inside and outside a silica core-PS shell microsphere illuminated by a Halogen lamp, Hg arc lamp, and monochromatic light of λ = 647 nm, respectively. For all the panels, *R*_*c*_ = 2.8 µm, *R*_*sh*_ = 3.0 µm, *ω*_*0*_ = 3 µm, and *n*_*m*_ = 1.0. The wavelength-dependent refractive indices of the PS and silica are estimated using Eqs. (34 and 35), respectively, and the obtained values are used for these simulations.

**Figure 13:**
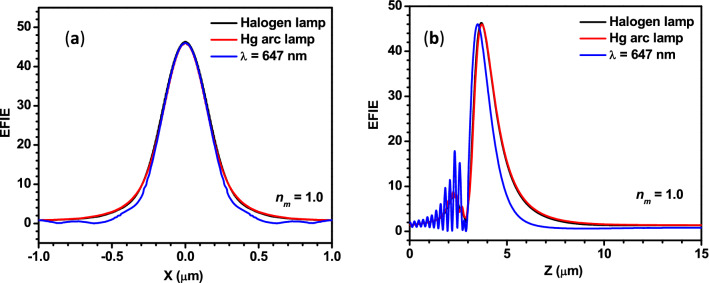
The variation in the EFIE along the transversal and longitudinal axes of the PNJs are shown in panels (**a**,**b**), respectively. For both the panels, *R*_*c*_ = 2.8 µm, *R*_*sh*_ = 3.0 µm, *ω*_*0*_ = 3 µm, and *n*_*m*_ = 1.0.

The theoretical investigation is extended for studying the PNJs of core–shell microspheres illuminated by polychromatic light. Panels (a) and (b) of Fig. 12 show the EFIE distribution of single silica core-PS shell microspheres obtained using the spectra of Halogen lamp and Hg arc lamp, respectively. From these two panels, it is clear that only one PNJ is observed in all the cases due to the thin shell. In general, several short and elongated PNJs are expected in the case of a thicker shell on a large sphere^59^ or multi-shells on a solid sphere^70^ due to multiple focusing and defocusing of the incident light. The *η*_*max*_ value found in the present case is lower than that obtained in the case of a solid PS microsphere of *R*_*s*_ = 3 µm. In the above sections, it is mentioned that the characteristic parameters of the PNJs of single solid microspheres obtained using the spectra of Halogen and Hg arc lamps are close to those obtained with the monochromatic light of λ = 655 nm. To find out the suitable wavelength of monochromatic radiation (*λ*_*mono*_) for the present case also, the EFIE distribution is plotted inside and outside the core–shell microspheres illuminated by monochromatic radiation of different wavelengths. From Figs. 12 and 13, it is clear that the *η*_*max*_, *W*_*eff,*_ and *L*_*eff*_ of the PNJs generated by core–shell microsphere under illumination with polychromatic radiation and monochromatic radiation of λ = 647 nm are very close. From these results, we can also conclude that one can use the characteristic parameters of the PNJ of core–shell microspheres (having thin shells) obtained with monochromatic light of *λ* = 647 nm to interpret the experimental results obtained with the PNJs of core–shell microspheres generated by polychromatic light from Halogen and Hg arc lamps.

**Figure 14:**
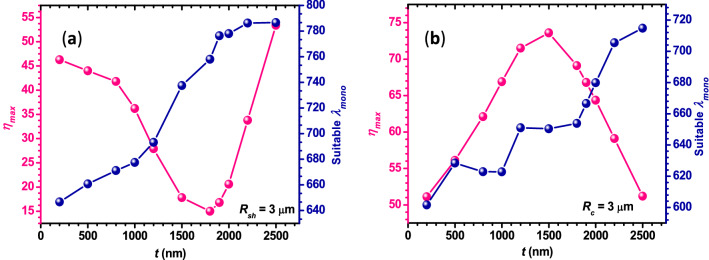
The pink curves in both the panels show the variation of *η*_*max*_ with *t* value in the case of polychromatic light from the Halogen lamp. The blue curves in both panels represent the variation of suitable λ_*mono*_ with the *t* value. For panels (**a**,**b**), the *t* value is varied by fixing the *R*_*sh*_ and *R*_*c*_ value, respectively. For all cases, *ω*_*0*_ = *R*_*sh*_ and *n*_*m*_ = 1.0.

The effect of shell thickness (*t*) on the characteristic parameters of the PNJs is also investigated theoretically. The pink curve in panel (a) of Fig. 14 represents the variation of *η*_*max*_ with the *t* value in the case of polychromatic radiation from the Halogen lamp. Here the *t* value is increased by fixing the value of *R*_sh_ at 3 µm and varying the *R*_*c*_ value from 2.8 to 0.5 µm. In this case, the value of *η*_*max*_ is found to decrease upon increasing the *t* value till 1800 nm. Beyond this *t* value, *η*_*max*_ is found to increase. For each *t* value, the suitable *λ*_*mono*_ has been found to generate the PNJ which has the characteristic parameters close to those obtained in the case of polychromatic radiation and mentioned in the same panel (blue color curve). In this case, the suitable *λ*_*mono*_ value is found to show an overall increase with *t.* When the *t* value is varied by fixing the *R*_c_ value at 3 µm and varying the *R*_*sh*_ value from 3.2 to 5.5 µm, the *η*_*max*_ is observed to follow the reverse trend in the case of polychromatic illumination [panel (b) of Fig. 14] in contrast to the previous case [panel (a)]. However, the suitable *λ*_*mono*_ value is again found to increase overall with the *t* value.

## Conclusions

The analytical equations which are useful to understand the characteristic parameters of the PNJs of single microspheres under polychromatic illumination are given in this paper. For the first time, a theoretical investigation is carried out on the PNJs generated using the emission spectra of Halogen lamp, SC source, white LED, and Hg arc lamp. For better comparison, the PNJs are also generated theoretically using monochromatic light of different *λ* values. The *η*_*max*_, *W*_*eff,*_ and *L*_*eff*_ of the PNJ obtained for all the polychromatic sources are slightly different due to the variation in the emission spectra. Relatively, the *η*_*max*_ is little larger, *W*_*eff*_ and *L*_*eff*_ values are slightly smaller in the case of white LED. These are found to be opposite in the case of SC source. The characteristic parameters of the PNJs of microspheres under polychromatic illumination are found sensitive with the *R*_*s*_ and *n*_*m*_ as in the case of monochromatic illumination. More importantly, the characteristic parameters of the PNJs of solid microspheres obtained for all polychromatic light sources are found close to those observed for the monochromatic light of *λ* which is near to the central wavelength of the polychromatic light sources. This means one can use the characteristic parameters of the PNJ of the solid microspheres observed with monochromatic light with suitable *λ* to interpret the experimental results obtained with the PNJs generated by polychromatic light from the above-mentioned sources.

In the case of core–shell microspheres, the characteristic parameters of the PNJs obtained with polychromatic light are sensitivity to the *t* value as in the case of monochromatic illumination. In addition, the suitable *λ*_*mono*_ values are found to increase with *t* values. We believe that the observations reported here are very useful for the researchers because generating PNJs numerically using monochromatic light is simple but the generation of PNJs numerically using polychromatic light is a difficult task and it demands a sophisticated computing facility.

## Supplementary Information

Supplementary Information.
