# Supplementary material for: Characteristic parameters of photonic nanojets of single dielectric microspheres illuminated by focused broadband radiation
Source: Sci Rep. 2022 Jan 7;12:173. doi: 10.1038/s41598-021-03610-3 (PMC8741904; doi:10.1038/s41598-021-03610-3)
Supplement: Supplementary file 1 — Supplementary Information. [file 41598_2021_3610_MOESM1_ESM.docx]

**Supplementary Information**

**Characteristic parameters of photonic nanojets of single dielectric microspheres illuminated by focused broadband radiation**

Amartya Mandal^1,^^†^, Pragya Tiwari^1,†^, Paul K. Upputuri^2^, Venkata R. Dantham^1,*^

^1^Department of Physics, Indian Institute of Technology Patna, Bihar, India – 801103

^2^School of Chemical and Biomedical Engineering, Nanyang Technological University, 62 Nanyang Drive, Singapore 637459

^†^Equal contribution

*Corresponding author: [dantham@iitp.ac.in](mailto:dantham@iitp.ac.in)

Fig. S1 shows the electric field intensity enhancement (EFIE) of the photonic nanojets (PNJs) generated by single solid silica microspheres. The EFIE distributions due to the illumination of monochromatic (λ = 634 nm) as well as polychromatic radiation (by Halogen lamp) are shown in the figure.

To make a comparison between the two outputs (Panels (a) and (b)), the variation in the EFIE along the transversal and longitudinal axes of the PNJs are also plotted and shown in the panel (c) and (d), respectively.

As mentioned in the manuscript, one can use the characteristic parameters of the PNJ obtained with monochromatic light of λ = 634 nm to interpret the experimental results obtained with the PNJs generated from silica microsphere using the polychromatic light from Halogen lamp.

| 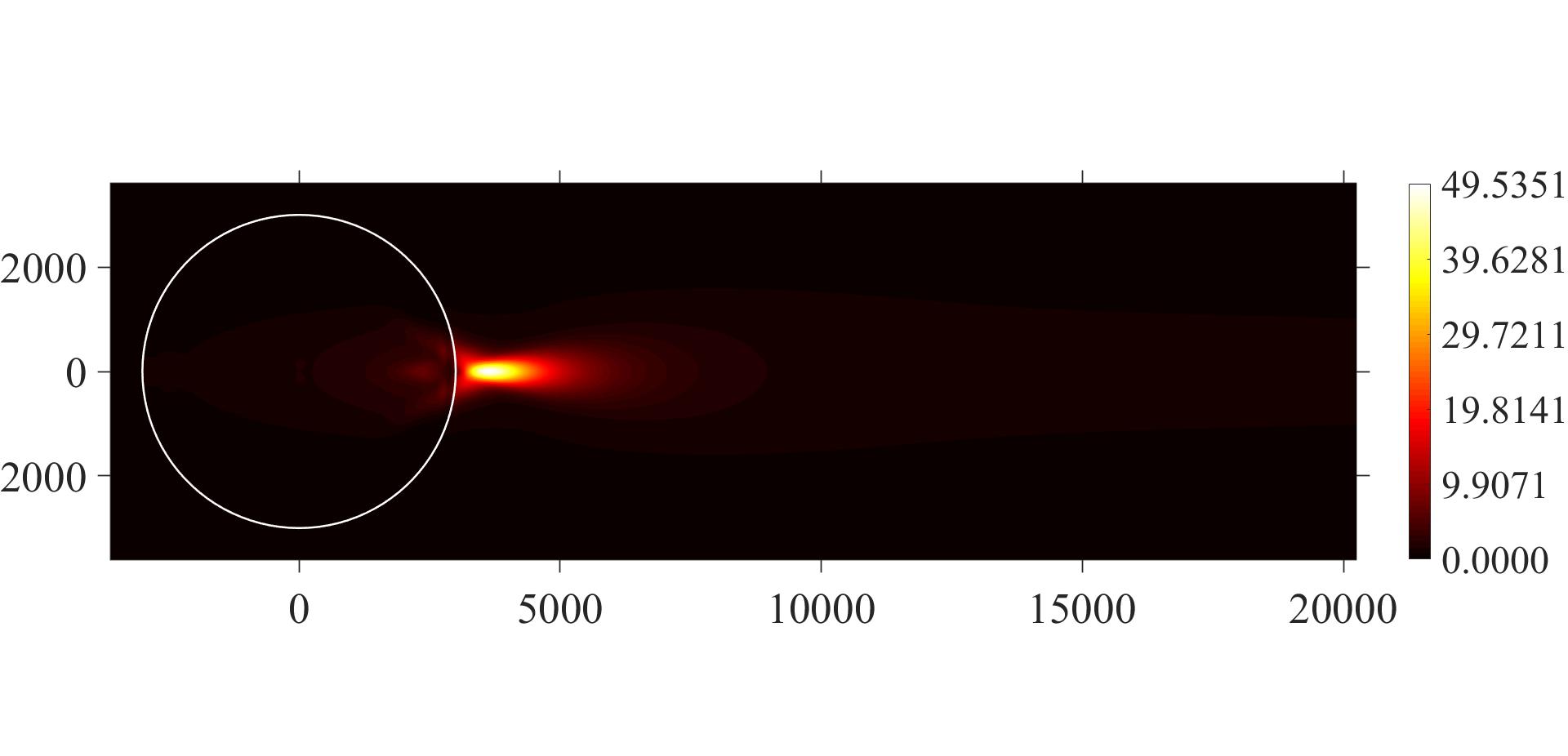  **Halogen lamp**  (**a**)  **2**  **0**  **2**  **X (µm)** | |
| --- | --- |
| 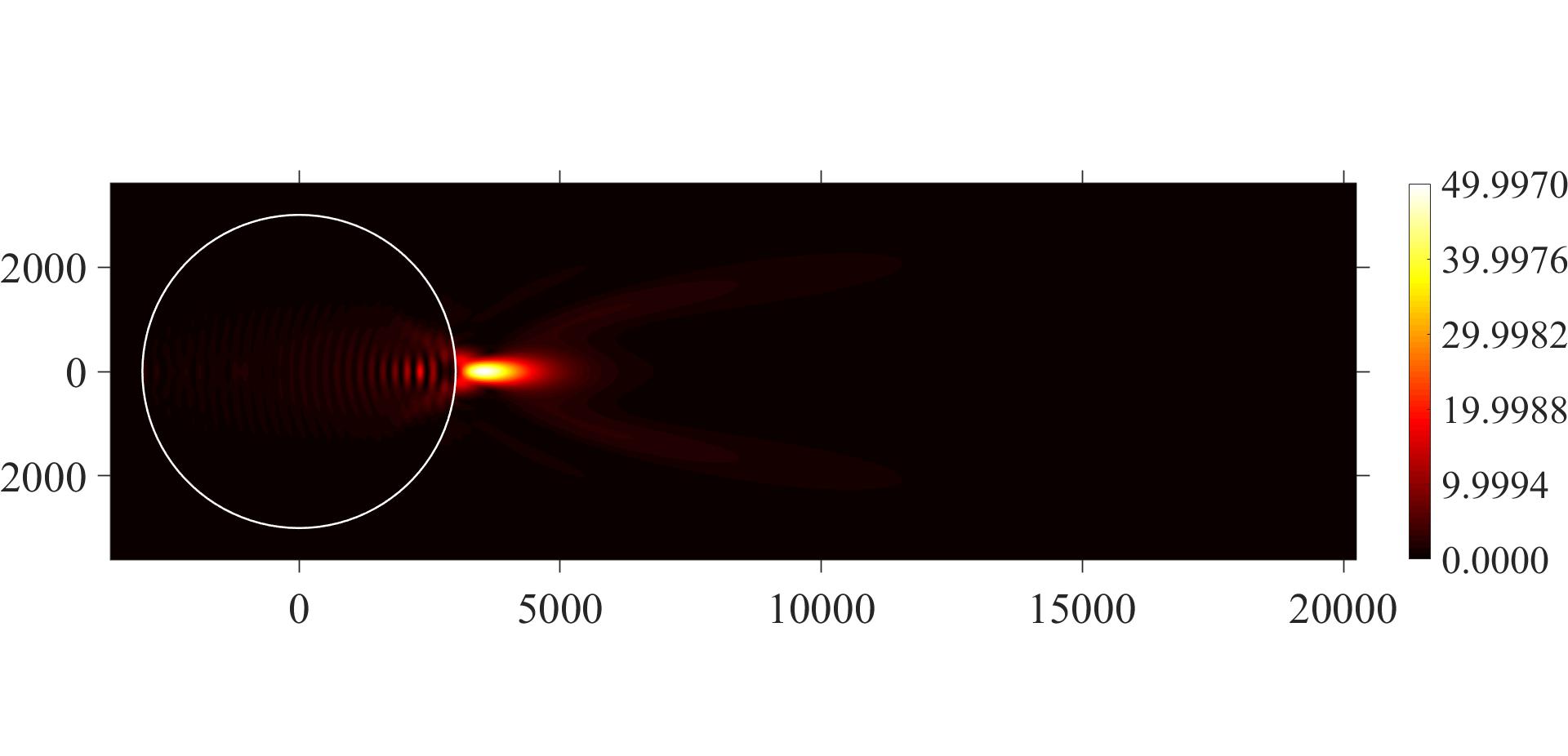  (**b**)  ***λ* = 634 nm**  **2**  **0**  **2**  **0**  **20**  **15**  **10**  **5**  **X (µm)**  **Z (µm)** | |
|   (**c**) |   (**d**) |

**Figure S1:** Panels (a) and (b) represent the EFIE distribution inside and outside a silica microsphere illuminated by a Halogen lamp and monochromatic light, respectively. The plots for variation in the EFIE along the transversal and longitudinal axes of the PNJs are shown in panels (c) and (d), respectively. For all panels, *R_s_* = 3 µm*, ω*_0_ = 3 µm, and *n_m_* = 1.0. The wavelength-dependent refractive indices of silica are taken from Ref. [66].

**Reference**

[66] Malitson, I. H. (1965). Interspecimen comparison of the refractive index of fused silica, J. Opt. Soc. Am. 55, 1205-1208.
